# Rare Genetic Variants in Jewish Patients Suffering from Age-Related Macular Degeneration

**DOI:** 10.3390/genes10100825

**Published:** 2019-10-18

**Authors:** Nadav Shoshany, Chen Weiner, Margarita Safir, Adi Einan-Lifshitz, Russell Pokroy, Ayala Kol, Shira Modai, Noam Shomron, Eran Pras

**Affiliations:** 1 The Matlow’s Ophthalmo-Genetics Laboratory, Department of Ophthalmology, Shamir (formerly Assaf-Harofeh) Medical Center, Zerifin 70300, Israel; drshoshany@gmail.com (N.S.); c.cweiner@gmail.com (C.W.); adi.einan@gmail.com (A.E.-L.); pokroy@gmail.com (R.P.); ayalak1977@gmail.com (A.K.); eranpras@gmail.com (E.P.); 2 Sackler Faculty of Medicine, Tel Aviv University, Tel Aviv 69978, Israel; nshomron@tauex.tau.ac.il; 3 Variantyx Inc., Framingham, MA 01701, USA; shira.modai@variantyx.com

**Keywords:** degeneration, genetics, macula, WES (whole-exome sequencing)

## Abstract

Purpose: To identify rare genetic variants in early age-related macular degeneration (AMD) utilizing whole-exome sequencing (WES). Methods: Eight non-related early-AMD families of different Jewish ethnicities were ascertained. Initial mutation screening (phase-1) included common *complement factor-H* (*CFH*) p.Y402H; and *age related maculopathy susceptibility 2* (*ARMS2*) p.A69S; and rare variants *complement factor-I* (*CFI*) p.V412M; and *hemicentin1* (*HMCN1*) c.4163delC identified previously in our population. Four families, whose initial screening for the aforementioned variants was negative, underwent WES (phase-2). Bioinformatics filtering was based on functionality (from a panel of 234 genes with proven or presumed association to AMD); predicted severity; and frequency (rare variants with minor allele frequency <1%). When applicable, further screening for specific rare variants was carried out on additional cases of similar ethnicities and phenotypes (phase-3). Results: Phase-1 identified three families carrying *CFI* p.V412M mutation. WES analysis detected probable disease-related variants in three out of the remaining families. These included: a family with a variant in *PLEKHA1* gene p.S177N; a family with previously reported variant p.R1210C in *CFH* gene; and two families with the *C3* p.R735W variant. Conclusions: Rare, high-penetrance variants have a profound contribution to early-AMD pathogenesis. Utilization of WES in genetic research of multifactorial diseases as AMD, allows a thorough comprehensive analysis with the identification of previously unreported rare variants.

## 1. Introduction

Age-related macular degeneration (AMD), a multifactorial disorder of the central retina, is the commonest cause of vision loss among the elderly in the developed world [1]. In most cases, AMD prevalence rises dramatically in the eighth decade; however, some patients exhibit early onset of disease manifestations and rapid progression (e.g., early-AMD) [2].

Environmental risk factors associated with AMD progression, such as smoking [3], fail to account for the phenotypic diversity of the disease. 

Two decades of research unraveled a complex influence of a handful of genes on disease pathogenesis. Genome-wide association studies (GWAS) have initially discovered the role of the common complement factor-H (*CFH*) p.Y402H variant (rs1061170) as an AMD risk factor [4]; followed by many other variants in complement system genes *CFI*, *C3*, *C2*, and factor-B [5,6,7]. Additional common-variants in the *PLEKHA1/ARMS2/HTRA1* complex, located in chromosome 10q region, including p.A69S (rs10490924) in age-related maculopathy susceptibility 2 (*ARMS2*) gene, have been reported. The independent effect of each variant in this locus is constantly debated, as the proximity of the involved genes in this complex hinders demonstration of linkage disequilibrium [8,9,10].

Despite the role of the aforementioned ‘common’ alleles in elevated disease risk, they account for only part of the total genetic load [11]. Rare variants with minor allele frequency (MAF) of less than 5%, usually undetected by GWAS, probably fill heritability void of various common diseases [12]. For example, rare *CFH* variants were found to associate with highly penetrant, early onset aggressive AMD [13]. 

The complement system, composed of over forty factors and regulators, has a pivotal role in AMD pathogenesis. *CFH* and complement factor I (*CFI*) are important regulators of complement activation. Unregulated activation of the alternative pathway, caused by absence of such regulators or compromised binding ability to target sites, allows tissue destruction by prolonged inflammation. In AMD, inflammatory damage to Bruch’s membrane is presumed to be an important insult. Similarly, when focal complement dysregulation involves the glomerular basement membrane, atypical hemolytic-uremic syndrome (aHUS) occurs. Interestingly, both disorders are associated with complement system gene mutations [14].

Identification of rare variants utilizing whole exome sequencing (WES) in AMD, as well in many other common diseases, is constantly on the rise. Examples include *CFH* p.R53C and p.D90G [15], *CFH* p.P503A [16]; *C3* p.K155Q [17]; and two rare variants reported by our group—*CFI* p.V412M and *HMCN1* c.4162delC [18].

In the current study, we utilized WES to uncover pathogenic variants in early-AMD families. Application of severity prediction tools on identified variants allowed the identification of rare variants, demonstrating the power and relevance of WES in this domain.

## 2. Methods

### 2.1. Patients and Clinical Evaluation

The study was approved by the institutional review board and informed consent has been obtained from all participants. 

Patients were identified at the retina clinic at “Assaf Harofeh” medical center, Zerifin, Israel. All index patients, in their seventh decade or earlier, exhibited early-AMD, with high incidence of geographic atrophy (GA) or choroidal neovascularization (CNV) with poor treatment response (progression despite conventional anti- vascular endothelial growth factor, VEGF, therapy). A positive family history of macular degeneration (when available) or visual impairment with vertical transmittance, consistent with autosomal dominant mode of inheritance, has been documented for most patients. For each index patient, a relative with similar disease characteristics or retinal findings was recruited. When no affected relatives were available, an unaffected relative was included for reference.

Clinical evaluation included a comprehensive ophthalmic examination as previously described [18]. This study was approved by the Institutional Review Board of Assaf Harofeh Medical Center, and adhered to the tenets of the Declaration of Helsinki, code 18-06.

### 2.2. Molecular Studies 

Laboratory work included the following consecutive phases:

(1) Mutation screening for previously reported rare and common variants in our population.
(2) WES and bioinformatics analysis.
(3) Screening an in-house cohort for more cases carrying the identified new variant.

### 2.3. Mutation Screening and Sanger Sequencing (Phase-1)

Blood samples were drawn from index patients and relatives. DNA was extracted using a commercial kit (Gentra System Inc., Minneapolis, MN, USA). As outlined above, Sanger sequencing of selected amplicons was carried out at first, in order to determine whether participants carry previously reported rare variants in the Israeli population (*CFI* p.V412M and (H*MCN1* c.4163delC). 

Patients were also assessed for the status of the most common AMD-related variants—*CFH* p.Y402H and *ARMS2* A69S—in order to evaluate their contribution (when applicable).

### 2.4. Whole Exome Sequencing and Bioinformatic Analysis (Phase-2)

Whole exome sequencing was carried out by a certified NGS laboratory (Macrogen, Rockville, USA), on a pair of DNA samples from each early-AMD family as described before [18]. In each affected family, samples were drawn from the proband (early-AMD case) and relatives who agreed to participate in the study (including clinical and genetic examinations). When applicable, we preferred to include as the second sample cases with a definite phenotype (either early-AMD case or unaffected). To focus our search for deleterious variants, a panel of 234 genes with known association to normal retinal structure and function, retinal pathologies, complement system, angiogenesis, and lipid metabolism, were defined. 

Rare variants were identified using data from dbSNP135 (Database of Single Nucleotide Polymorphisms (dbSNP) [19]. Bethesda (MD): National Center for Biotechnology Information, National Library of Medicine), the 1000 Genomes Project [20], the National Heart, Lung, and Blood Institute (NHLBI) Exome Sequencing Project Exome Variant Server (Exome Variant Server, NHLBI GO Exome Sequencing Project (ESP). Seattle, WA [21], and the genome aggregation database (gnomAD) [22]. In addition, frequency of selected rare variants was tested in an in-house database of 1500 sequenced individuals of different Israeli ethnicities including approximately more than 300 Ashkenazy Jews; 100 North-African Jews, 60 Oriental Jews, and 1000 Israeli exomes of unspecified origin). Variants with an allele frequency >1% in any of these databases were excluded from further analysis.

Variants were classified according to predicted protein effects with PolyPhen [23] and SIFT [24]. Annotation and analysis of rare variants was made using Annovar [25]. Variants predicted to be deleterious by more than five prediction tools, or those that resulted in loss-of-function mutation, were considered as candidate variants. Deleterious mutations and variants of unknown clinical significance were subjected to extensive literature and database searches to determine their relatedness to AMD. 

### 2.5. Further Screening of an In-House AMD Cohort for a Presumed Pathogenic Variant (Phase-3)

Deleterious variants with relevance to AMD according to WES results underwent further Sanger sequencing for verification, segregation-analysis within the extended family (when available), determination of allele frequencies, and screening an in-house cohort of 60 AMD cases (40 Ashkenazi Jews and 20 Tunisian Jews) for the newly identified variant. 

## 3. Results

Altogether, our analysis yielded a probable disease-related variant in 7/8 families (Families 1–8), three of which with *CFI* p.V412M mutation, as described below (Table 1). The carrier status of the common variants *CFH* p.Y402H and *ARMS2* p.A69S are shown as well (Table 2). At least one proband in each family manifested early-AMD featuring before 65 years. Frequently, other siblings who carry the variant and manifest the phenotype were also found. 

As described in detail below, in Phase-1 we have identified by mutation screening three additional families (Families 4, 5, 8) of Tunisian (North-African) Jews ancestry who carry the previously reported *CFI* p.V412M mutation. In Phase-2, four pairs of subjects from unrelated early-AMD families (Familes 1–3, and Family 7) underwent WES that ended with the identification of three probable disease-causing mutations (Table 1). Screening an in-house cohort comprised of 60 AMD (Phase-3) for these mutations identified another Jewish Ashkenazi family (Family 6) with *C3* p.R735W. No candidate rare mutation found for Family 7.

### 3.1. Mutation Screening for Previously Described Mutations in CFI and HMCN1 Genes (Phase-1)

#### Families 4, 5, and 8 with CFI p.V412M (Figure 1A–G) 

**Figure 1:**
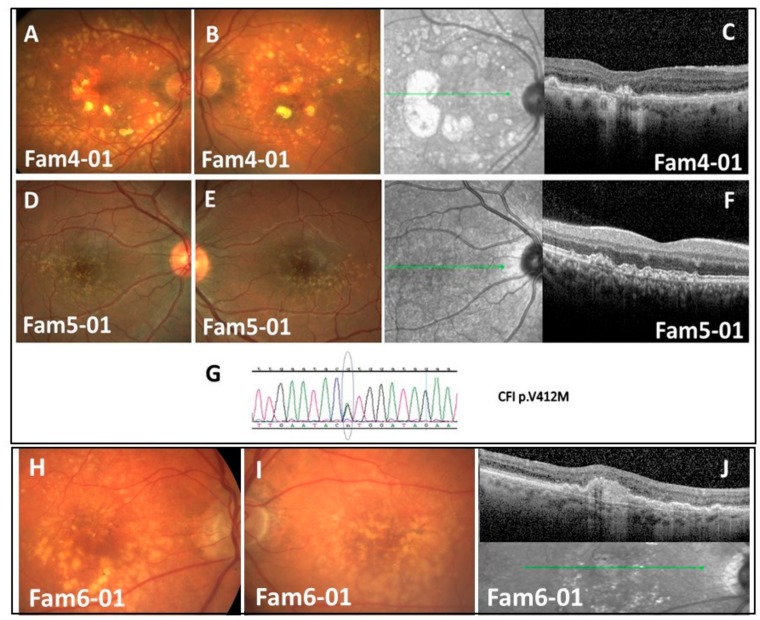
Clinical findings and sequencing results for families 4,5 with CFI p.V412M and family 6 with C3 p.R735W. (**A**,**B**) Color fundus images of Fam4-01—demonstrating large confluent drusen extending beyond vascular arcades and patches of RPE atrophy; (**C**) SD-OCT image of Fam4-01—demonstrating central atrophy and scarring; (**D**,**E**) Color fundus images of Fam5-01—demonstrating bilateral drusen; (**F**) SD-OCT image—demonstrating drusen and drusenoid PEDs; (**G**) Family 4 and Family 5 sequencing results—CFI p.V412M; (**H**,**I**) Color fundus images of Fam6-01—demonstrating multiple drusen and pigmentary changes; (**J**) SD-OCT image of Fam6-01—demonstrating drusen, drusenoid PEDs, and sub-retinal fibrosis. Wild-type (WT)/Mutation (M).

Screening Families 4, 5 of Tunisian (North-African) Jews ancestry, for the previously described mutations by our group (*CFI* p.V412M and *HMCN1* c.4162delC) [18], revealed that affected individuals are heterozygous carriers for *CFI* p.V412M (Figure 1G). This discovery provides additional evidence for the pathogenicity of this mutation which demonstrates till now full- penetrance and segregation with early-AMD in five families, and 15 patients. The proband of family-4 was a late-7th decade female (Fam4-01) with severely impaired visual acuity (20/200 in both eyes) and bilateral central geographic atrophy (Figure 1A,B); whereas in Family 5, the proband (Fam5-01) presented with exceptionally early-onset presentation (to the best of our knowledge the earliest description ever) of drusen in both of his eyes at 21 years only (Figure 1D–F). This asymptomatic young adult was discovered by chance during fundus examination as part of his general medical assessment for mild-hypertension. No other family members were available for examination, but medical history unraveled that the proband’s father who suffered from end-stage renal disease was treated in a different facility with bilateral intra-vitreous anti-VEGF injections. The patient in family 8 had advanced AMD.

### 3.2. WES Analysis (Phase-2) and Screening More Families for an Identified Mutation (Phase-3) 

#### 3.2.1. Family-1 with CFH p.R1210C (Figure 2A–E)

Affected sib of this family was a woman of Jewish Ashkenazi ancestry in her seventh decade (Fam1-01), with severe visual impairment secondary to advanced bilateral atrophic AMD (Figure 1B–D). Best corrected visual acuity (BCVA) was limited to finger counting. Over a year of follow-up, her central GA enlarged in both eyes, without evidence for CNV. Although no other family members had visual complaints or characteristic fundus changes, the rapid deterioration and extent of atrophy warranted inclusion of the patient in our study. The proband’s asymptomatic and phenotypically unaffected sister (Fam1-02) has been included for comparison.

At the end of the filtration process of WES results, a single suspicious heterozygous rare variant in *CFH* gene was identified; c.C3628T; p.R1210C (rs121913059). In addition to its association with aHUS [26], it has been described as a risk factor for advanced AMD, and GA in particular [27], exerting high penetrance (97.5%), and early onset [28].

The unaffected sister (Fam1-02) was found homozygous wild-type (WT) at this position, and had no other high-risk rare variants identified. Sanger sequencing of the proband’s asymptomatic offspring’s disclosed that (Fam1-03, Fam1-04) were also WT. The youngest (early fifth decade) son (Fam1-05) was heterozygous carrier for the variant, with preserved vision (BCVA 6/8.5 in both eyes) and no fundus abnormalities.

#### 3.2.2. Family-2 with PLEKHA1 P.S177N (Figure 2F–J)

Family-2 consisted of four siblings of Jewish Syrian (Oriental) ancestry with severe vision loss. Fundus examinations of all of them demonstrated extensive geographic atrophy and macular scaring. Other families were unavailable for examination. Two DNA samples (Fam-2 01 and 02) underwent WES which filtering’s process identified in both samples a single rare heterozygous variant *PLEKHA1* c.G530A;p.S177N (rs142473166). All severity prediction tools classified it as probably-deleterious. 

Subsequent analysis of DNA samples from the remaining affected sisters (Fam-2 03 and 04) revealed that they also carry heterozygously this rare variant; making complete segregation of the mutation with advanced-AMD in this family. Further inspection of WES data from this family reassured that the identified *PLEKHA1* variant segregates with WT alleles of both *ARMS2* and *HRTA1* genes in all sibs, thereby strengthening the arguments regarding the pathogenicity of this rare variant.

#### 3.2.3. Family-3 with C3 p.R735W; ABCA4 p.G1931E; CFI p.K441R (Phase-2)

**Figure 2:**
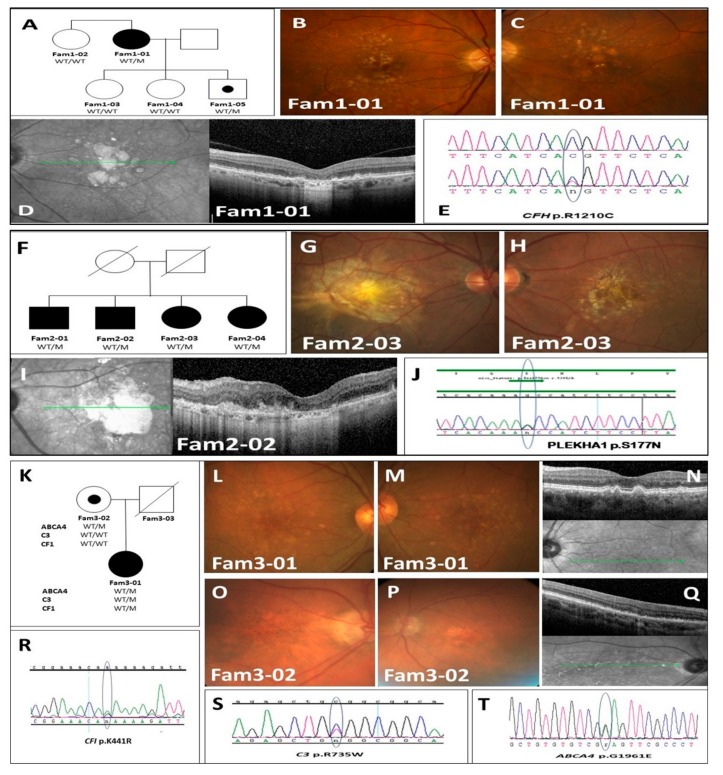
Family 1–3 pedigrees, clinical findings and sequencing results. (**A**) Family 1 pedigree; (**B**,**C**) Color fundus images of Fam1-01 demonstrating drusen and hypopigmentation secondary to atrophy; (**D**) SD-OCT image of Fam1-01—demonstrating subfoveal atrophy; (**E**) Family 1 sequencing results—*CFH* p.R1210C; (**F**) Family 2 pedigree; (**G**,**H**) Color fundus images of Fam2-03—demonstrating drusen and pigment changes secondary to atrophy and scarring; (**I**) SD-OCT image of Fam2-02—demonstrating retinal disorganization and hydration; (**J**) Family 2 sequencing results—*PLEKHA1* p.S177N; (**K**) Family 3 pedigree; (**L**,**M**) Color fundus images of Fam3-01 demonstrating multiple drusen; (**N**) SD-OCT image of Fam3-01—demonstrating drusen and drusenoid PEDs; (**O**,**P**) Color fundus images of Fam3-02—demonstrating hyper and hypo-pigment macular changes; (**Q**) SD-OCT image of Fam3-02—demonstrating drusen and atrophic outer retinal changes; (**R**) Family 3 sequencing results—*ABCA4* p.G1961E; (**S**) Family 3 sequencing results—*C3* p.R735W; (**T**) Family 3 sequencing results—*CFI* p.K441R.

The proband in Family 3 (Fam3-01) was an Ashkenazi Jew woman in her seventh decade that manifested extensive large confluent drusen and retinal pigment epithelium (RPE) changes in both fundi, consistent with advanced-AMD (Figure 2L–N). Although BCVA was relatively preserved (6/9 in both eyes), her impressive clinical findings pointed to an aggressive disease course. 

Her mother (Fam3-02), in her late ninth decade with prolonged cigarette smoking history, had BCVA of 6/30 in both eyes and subtler retinal findings compatible with intermediate-AMD ((Figure 2O–Q). The proband’s father (Fam3-03) had passed over a decade prior to the study, and not included in the genetic analysis. 

WES performed on both subjects, identified, in Fam3-01, three rare variants of known risk for AMD (and HUS): *ABCA4* p.G1961E (rs1800553) [29]; *C3* p.R735W (rs117793540) [30]; and *CFI* p.K441R (rs41278047) [31,32] (Figure 2R–T). Only *C3* p.R735W variant was identified in 3 of 1500 genomes (of subjects with unknown disease status) of the Israeli exome database, and also was found in one out of the 40 Ashkenazi Jews DNA samples of AMD patients of our in-house cohort. All three mutations were identified heterozygously in the proband (Fam3-01), while her mother Fam3-02 carried the *ABCA4* p.G1961E change only which has an attributed risk for AMD of 3.22 fold among heterozygous carriers [33].

#### 3.2.4. Family-6 with C3 p.R735W (phase-3) (Figure 1H–J)

Mutation screening had identified in one out of 40 DNA samples from our in-house cohort (aforementioned) the *C3* p.R735W variant. This sample belongs to an Ashkenazi Jewish patient (Family 6-01) with advanced-AMD, harboring large drusen and extensive RPE changes (Figure 1H–J). The patient’s 50-year old only son had an unremarkable fundus appearance and was negative for the variant. According to EUGENDA database [30], this variant elevates the risk for advanced-AMD by a 17.4-fold.

#### 3.2.5. Family 7

Family 7 consisted of a Jewish Ashkenazi couple in their seventh decade, with reported consanguineous marriage (first degree cousins), and intermediate AMD findings. Bioinformatics analysis of the sequencing data of both subjects provided no rare variants with high predicted severity in the entire AMD panel. 

#### 3.2.6. Family 8

Family 8 consisted of a 41-year-old female of Tunisian (North-African) Jewish descent and a phenotype of early AMD in both eyes. Family history was remarkable for a father with diagnosis of blindness (no additional information was available) and an uncle with renal failure. No additional family members were available for examination. Bioinformatics analysis revealed heterozygous state for p.V412M mutation of *CFI* gene.

## 4. Discussion

### 4.1. Patients and Gene Selection

The power of WES in identifying rare variants associated with complex diseases is gaining recognition in the current era. In this study, we demonstrate the advantage of utilizing this technology for the identification rare deleterious variants in early-AMD families. Selection of patients manifesting a phenotypic triad of: early disease onset; phenotypic aggressiveness; and positive family history, enabled a relative high detection yield of 85% (7/8).

Candidate genes were meticulously selected to constitute a broad panel of genes thorough relevant medical literature review and relevance to AMD pathogenicity. Of special interest were complement system genes, hosting most of the known variants with associations with AMD thus far. Inclusion of genes with known "protective" variants (e.g., *CFH* p.N1050Y, *PELI3* p.A307V) [34] in our panel required no further action, since identified protective variants have been reported in the complement system, extracellular matrix, and lipid metabolism genes; all of which were included originally as risk-genes. No protective variants were identified in our study, presumably due to a relatively small sample size and phenotypic aggressiveness of the cases. Undoubtedly, additional genes involved in pathogenesis are yet to be discovered. Future re-analysis of the sequencing data could potentially identify in Family 7 a novel gene which has not been included in our AMD gene panel.

### 4.2. Rare Variants

Among the six rare variants identified in the current study, four have previously been described in association with AMD in other populations (*ABCA4* p.G1961E, *CFI* p.K441R, *C3* p.R735W, and *CFH* p.R1210C), and one variant has been reported in Tunisian Jews (*CFI* p.V412M) [18]; and the *PLEKHA1* p.S177N variant has been reported in only two cases of familial AMD [32,35]. In-line with previous knowledge on disease pathophysiology, most variants occurred in complement-system genes. We are aware of the risk of a possible selection-bias, caused by inclusion of a wide selection of complement system genes in our panel. However, the pathophysiologic rationale and existing proof of complement system involvement in previous AMD studies support the incorporation of complement genes as pivotal components of any AMD gene-panel.

To date, *CFH* p.R1210C (identified in Family 1) is the most recognized enhancer for AMD. Heterozygous carriers were reported to harbor a 20-fold increase in disease risk [27]. Fam1-01 exhibit large atrophic changes, which have also been described among carriers [36]. The variant has also been described in association with heritable forms of aHUS and primary glomerulonephritis [13], but our patients had normal renal function.

Like factor-H, CFI is also an important complement regulator and variants of high predicted severity were found more prevalent in AMD patients. The detected variant *CFI* p.V412M, manifests till now full-segregation with AMD in 15 patients from five separate Tunisian Jews families (Fam-1 and Fam-3 described previously [18] and families 4, 5, 8 presently). According to bio-informatics prediction tools this variant has high deleterious effect on factor-I’s functionality and complement regulation. This calculated risk is in-line with the observed aggressiveness of the phenoptype which exerts very high penetrance, and much earlier onset as compared with popular AMD. To the best of our knowledge, two patients carrying this variant (Fam5-01 described presently and Fam3-01 previously) exhibit the earliest description of AMD thus far at 21, and 24 years of age, correspondingly.

Interestingly, the three rare variants identified in Fam-3-01 patient (*CFI* p.K441R, *C3* p.R735W, and *ABCA4* p.E1961G) may exemplify a complex influence of multiple risk enhancers on overall phenotype.

*PLEKHA1* gene is part of the chromosome 10q26 locus with a well-recognized association to AMD [37]. Three genes at this region (*ARMS2* and *HTRA1* and *PLEKHA1)* are in a state of tight linkage-disequilibrium which furnishes a long lasting debate which of whom is functionally responsible for the strong association of this locus. *PLEKHA1* variants have formerly been suggested to account for a 5-fold increase the risk for AMD [38]. The *PLEKHA1* p.S177N variant (MAF = 0.001735) found in four siblings of Family 2, was classified as “probably deleterious” by bioinformatics prediction tools. Although the pathologic mechanism of *PLEKHA1* is yet to be unraveled, our study strengthens the arguments attributing a pathogenic role to PLEKHA1 in AMD. Not only does the variant segregate with aggressive-AMD within four members of the present family adding significance to previous descriptions [32,35] but the neighboring genes (*ARMS2* and *HTRA1*) were also found to carry the wild-type alleles only. Nonetheless, we could not exclude the possibility that the identified variant lies in tight linkage disequilibrium with other pathogenic change in the locus, such as regulatory elements or deletion, which escaped detection by WES.

### 4.3. Common Variants

Current hypothesis for disease-risk estimates suggests that common variants’ contribution may significantly be lower than rare variants. We have included two of the most recognized variants in our evaluation and found a compatible prevalence for both *CFH* p.Y402H, and *ARMS2* p.A69S, with the general population. The proband in family-1 who carried the deleterious *CFH* p.R1210C rare variant, had also carried heterozygous *CFH* p.Y402H and *ARMS2* p.A69S. Thus, the cumulative risk for early aggressive AMD in this patient can be attributable to both common and rare variants. Family 2 subjects were found homozygous WT for the respective loci, a fact that intensifies the presumed contribution of *PLEKHA1* p.S177N variant to disease risk. Family 7 subjects, in whom no rare variant was identified, were also homozygous WT carriers in both loci, thus implicating additional, hitherto undiscovered, AMD risk variants in genes not included in our panel. Additional work is therefore needed to expand the scope of genes and variants screened, in an effort to find the cause of their blinding disease.

### 4.4. Summary

The power of WES in unraveling genetic etiology of a common complex disease as AMD is demonstrated in our study. The identified mutations expand the repertoire of AMD-related mutations, intensify the hypothesis that in complex disorders the over-whole disease risk is influenced from multiple “insults” including rare-deleterious variants; and emphasize the power of NGS in their detection.

## 5. Conclusions

WES provides an inexpensive, efficient, and reliable method to uncover complex genetic collage. Further work is warranted to expand the scope of selected gene panels, and to allow better identification and annotation of variants, alongside association of different phenotypes to specific variants or haplotypes.

## Figures and Tables

**Table 1:** Rare variants of high predicted severity (initial WES (whole-exome sequencing) findings and subsequent findings in additional families).

Family	Ancestry	Analysis Method		Gene	c. Variant	p. Variant	Effect	Predicted Severity	Prevalence	Proband	Relative (Affectation Status +/−)
		WES	Mutation Screening								
1	Jewish Ashkenazi	+		*CFH*	c.3268C>T	p.Arg1210Cys	Nonsyn. SNV	High	0.000173	Het	WT Hom (−)
2	Jewish Syrian (Oriental)	+		*PLEKHA1*	c.530G>A	p.Ser177Asn	Nonsyn. SNV	High	0.001735	Het	Het (+)
3	Jewish Ashkenazi	+		*ABCA4*	c.5882G>A	p.Gly1961Glu	Nonsyn. SNV	High	0.0023	Het	Het (+) **
				*CFI*	c.1346A>G	p.Lys441Arg	Nonsyn. SNV	High	0.0009	Het	WT Hom (+) **
				C3*	c.2203C>T	p.Arg735Trp	Nonsyn. SNV	High	0.0005	Het	WT Hom (+) **
6			+								-
4	Jewish Tunisian (North African)		+	*CFI*	c.1234G>A	p.Val412Met	Nonsyn. SNV	High	0.000107	Het	Het (+)
5			+								
8			+								-

c. Variant = complementary DNA variant; p. Variant = protein variant; Nonsyn. SNV = Nonsynonymous single nucleotide variation; WT Hom = Wild-Type Homozygous; Het = Heterozygous carrier of the variant. ** In family-3 affected relative had milder AMD phenotype than the proband.

**Table 2:** Carrier status of common variants *CFH* p.Y402H and *ARMS2* p.A69S.

Family	Subject	*CFH* p.Y402H	*ARMS2* p.A69S
1	Proband	Het	Het
	Relative	WT Hom	Het
2	Proband	WT Hom	WT Hom
	Relative	WT Hom	WT Hom
3	Proband	WT Hom	WT Hom
	Relative	WT Hom	WT Hom
4	Proband	WT Hom	Het
	Relative	WT Hom	WT Hom
5	N/A		
6			
7			
8			

WT Hom = Wild-Type Homozygous; Het = Heterozygous carrier of the variant. Hom = Homozygous carrier of the variant.
